# Real-time predictive seasonal influenza model in Catalonia, Spain

**DOI:** 10.1371/journal.pone.0193651

**Published:** 2018-03-07

**Authors:** Luca Basile, Manuel Oviedo de la Fuente, Nuria Torner, Ana Martínez, Mireia Jané

**Affiliations:** 1 Public Health Agency of Catalonia, Barcelona, Spain; 2 Technological Institute for Industrial Mathematics (ITMATI), Campus Vida, Santiago de Compostela, Spain; 3 MODESTYA Group, Department of Statistics, Mathematical Analysis and Optimization, University of Santiago de Compostela, Santiago de Compostela, Spain; 4 Department of Medicine, University of Barcelona Barcelona, Spain; 5 CIBER Epidemiology and Public Health CIBERESP, Carlos III Health Institute, Madrid, Spain; Columbia University, UNITED STATES

## Abstract

Influenza surveillance is critical to monitoring the situation during epidemic seasons and predictive mathematic models may aid the early detection of epidemic patterns. The objective of this study was to design a real-time spatial predictive model of ILI (Influenza Like Illness) incidence rate in Catalonia using one- and two-week forecasts. The available data sources used to select explanatory variables to include in the model were the statutory reporting disease system and the sentinel surveillance system in Catalonia for influenza incidence rates, the official climate service in Catalonia for meteorological data, laboratory data and Google Flu Trend. Time series for every explanatory variable with data from the last 4 seasons (from 2010–2011 to 2013–2014) was created. A pilot test was conducted during the 2014–2015 season to select the explanatory variables to be included in the model and the type of model to be applied. During the 2015–2016 season a real-time model was applied weekly, obtaining the intensity level and predicted incidence rates with 95% confidence levels one and two weeks away for each health region. At the end of the season, the confidence interval success rate (CISR) and intensity level success rate (ILSR) were analysed. For the 2015–2016 season a CISR of 85.3% at one week and 87.1% at two weeks and an ILSR of 82.9% and 82% were observed, respectively. The model described is a useful tool although it is hard to evaluate due to uncertainty. The accuracy of prediction at one and two weeks was above 80% globally, but was lower during the peak epidemic period. In order to improve the predictive power, new explanatory variables should be included.

## Introduction

Influenza is one of the biggest public health challenges worldwide. In temperate countries, seasonal influenza is a matter of concern due to its attributable excess morbidity and mortality.[1] Demand for health services increases exponentially during the cold season, highlighting the need for efficiently-programmed health policies to coordinate all stakeholders involved in managing the epidemic.[2]

The main mitigating health action during influenza epidemics is the organization of the influenza vaccine supply in the at-risk population.[3] Other activities needed at a policy-maker level during the cold season are the deployment of resources to mitigate the effects of influenza (e.g. antiviral drugs) and the organization of healthcare centres.[4] Therefore, predictive mathematical models that provide advance information on the intensity of the epidemic are important preparative measures.[5]

Mathematical modelling in epidemiology is concerned with describing the spread of diseases and their effects. It requires a multidisciplinary approach, involving health sciences, mathematics, engineering and even sociology and philosophy, in order to achieve better understanding of the spread of infection and possible control strategies.[6]

Specific models have been designed for diseases such as measles,[7] rubella, chickenpox,[8] dengue fever,[9] whooping cough,[10] smallpox,[11] malaria,[12] HIV/AIDS [13] and others.[14] Most models take the basic reproduction number R0 of the causative agent, defined as the threshold quantity that determines when an infection can invade and persist in a new host population, as one of the main assets.[15] Disease-free equilibrium of the model is linearly stable if R0 < 1 and unstable if R0 > 1.[16]

There are various methods of forecasting influenza activity, depending on the data available and the type of model used.[17,18] The most frequently used models are those involving time series.[19] ARIMA models and lineal regression models are the most common technics for monitoring of influenza using time series data.[20]

The generalized least squares (GLS) technique applied to lineal regression models improves the model fit by adjusting correlated errors.[21] However, models that take geographical distribution and time evolution into account are probably the best for local prediction of the intensity of the epidemic and correct evaluation of differences between areas.[22,23] Functional data analysis is an interesting tool that allows geographical and time factors to be combined in a single measurement.[24,25]

The pattern of epidemic activity of seasonal influenza remains quite similar year after year and variations in intensity and duration depend on many factors, including temperature[26], humidity,[27] vaccine effectiveness,[3] human mobility,[28] influenza lineage, etc.

So having any meteorological data (temperature, humidity and irradiation) for different spatial points at the same time could be useful not only for anticipating weather events that may influence in influenza activity, but also for detecting geographical patterns for influenza distribution.

Epidemiological surveillance is a useful tool for the early detection and characterization of circulating viruses. Spain, like most European countries, has sentinel networks that monitor the circulation of influenza in real time. In Catalonia, the PIDIRAC network of sentinel physicians has reported daily ILI (Influenza Like Illness) activity in their ascribed population, weekly respiratory samples for virological testing and the characterization of influenza viral strains, since 1999.[29] The statutory reporting disease system (MDO) is another source of available data in Catalonia for influenza incidence,[30] and permits assessment of the geographical distribution of reported ILI cases and local ILI incidence rates.

Google Flu Trends was another tool that estimated influenza intensity,[31,32] but unfortunately is no longer updated, even though the historical series is available online.[33]

### Objectives

The objective of this study was to design a real-time model to predict weekly influenza incidence rates one or two weeks in advance by matching of PIDIRAC, MDO, Google Flu Trends and meteorological databases, and taking into account the geographical distribution of outbreaks in Catalonia during the 2015–2016 influenza season.

## Materials and methods

### Data sources

The influenza forecast was based on ILI incidence rates received weekly by the MDO system. ILIs are grouped by health region (Lleida, Tarragona, Terres de l’Ebre, Girona, Catalunya Central, Alt Pirineu, Barcelona) and weekly global and regional ILI incidence rates per 100,000 people from 2010–2011 season was available.

The following data sources are used to select explanatory variables to include in the model:

- MDO source: ILI incidence rate per 100,000 persons (**MDO_ILI**) and the same rate taking into account a Susceptible-Infectious-Recovered construct (SIR)[34] (**MDO_SIR**), assuming that each case cannot be infected more than once in the same season.
- Meteorological data source: data were obtained from the official climate service in Catalonia, Meteocat.[35] Daily values of mean temperature, humidity and sunlight for each region were included as explanatory variables for study in two ways: first, the last daily value of temperature, humidity and sunlight for week T was taken into account(**Temp**, **Irr**, **Hum**) and, secondly, a functional variable was created to represent with a single value the evolution of temperature, humidity and sunlight over the last 14 days, (**Temp_F**, **Irr_F**, **Hum_F**).
- PIDIRAC source: it represent the principal tool in Catalonia for the quantification of influenza morbidity and characterization of the circulating influenza virus. Every week, the overall influenza incidence rate and the rate for age-groups (0–4, 5–14, 15–64, >64) is provided by 60 strategically-distributed primary healthcare physicians, who. So the 4 Age-grouped rates (**Baby**, **Child**, **Adult**, and **Elderly**) and overall rate (**PIDIRAC**) are included.
- Laboratory data source: Sentinel physicians from PIDIRAC program also send blood samples to reference laboratory for analysis, which provides the percentage of influenza virus isolations (**ISO%**).
- Google Flu Trends source: a useful on-line database of geographic influenza activity, was available until August 2015, when updating ceased. The pilot study, which was made before the last update, took into account Google Flu Trends indexes for **Spain**, **Catalonia**, **Aragón**, and the Valencian Community (**Valencia**).

### Analysis

Both pilot test and real-time model were developed using R-Studio software [36] and the weekly report document was written in LaTeX using TexStudio software. [37]

Before introducing a real-time forecast model for the 2015–2016 season, a pilot study was made at the end of the 2014–2015 season in order to select the type of multivariate model that provided the best forecast and the explanatory variables to include in the model.

For every possible explanatory variable we created a time series with data of the last 4 seasons (2010–2011, 2011–2012, 2012–2013, and 2013–2014).

The first phase of the pilot study analysed the distance correlation [38] between all explanatory variables at week T and the observed ILI incidence rates from the MDO source at week T+1 and T+2. We also analysed distance correlation between each health region to evaluate differences in influenza activity between regions. In case of high correlations between regions (more than 0.9) we included in the model as explanatory variable the MDO_ILI rate of the region with highest correlation with studied region.

In the second phase of the pilot study, we searched and identified five types of mathematical models. We started with the traditional approach of time series, an Autoregressive moving average model (ARMA). In this case the selected model was a time series model with autoregressive (AR) (p = 1) and moving average (MA) (q = 1) components. This model was adjusted using seasonality S = 52 weeks per year. [19] Another traditional way to forecast is using a multivariate linear regression model (LM). In this case, the limitations are the correlated residuals.[20] We could avoid this problem with a generalized least square regression model (GLS). This approach allows correlated residuals to be adjusted and includes an AR(p = 1) component.[21] Taking into account that a meteorological functional variable provides more temporal information about weather than a simple one, we included the functional component to LM and GLS models, obtaining the functional linear regression model (FLM) [24] and the functional generalized least squares regression (FGLS) (S1 Appendix). [25]

Arima() and lm() functions from “stats” R-package has been used to make predictions with ARMA and LM, gls() function from “nlme” R-package has been used for GLS, fregre.lm () and fregre.gls () from “fda.usc” R-package has been used for FLM and FGLS.

Firstly we calculated the Akaike Information criterion (AIC) to compare the relative quality of the 5 fitted models and quantify the information loss for every model during the entire available period (from week 40^th^ of 2010 to week 20^th^ of 2015). Then we used these 5 models to simulate a weekly prediction from week 40^th^ of 2014 to week 20^th^ of 2015 of the ILI incidence rate one (T+1) and two (T+2) weeks after the week T. Differences in predictions between models are tested by comparing the mean squared error (MSE). The model with the lowest MSE at one and two weeks was selected for the real-time forecast in the next season.

Before the 2015–2016 season starts, we identified five levels of intensity (baseline, low, medium, high, very high) for this season according to the Moving Epidemic Method (MEM),[39] and based on MDO data from the last five seasons (from 2010–2011 to 2014–2015), which helped to identify the epidemic period and influenza intensity during the 2015–2016 season.

During the 2015–2016 season, explanatory variables were included in the model following a stepwise selection: for every data source we included the variable that presented highest distance correlation for T+1 in the pilot study. In case of missing data during any week, the model automatically selected the second variable from the same source with highest correlation. Weekly, explanatory variables were updated and the model was loaded, obtaining predicted rates and the corresponding 95% confidence intervals. In addition, we published on Tuesday of every week a report that graphically and numerically represented observed rates for the last week (T) and predicted incidence rates for the actual week (T+1) and the next one (T+2) for each health region and globally.[40]

To evaluate the quality of predictions we created two index: The confidence intervals success rate (CISR) and the Intensity level success rate (ILSR). The CISR for T+1 or T+2 is the percentage of observations that remained inside the confidence intervals of rates predicted one or two weeks previously. The ILSR for T+1 or T+2 is defined as the percentage of observations that match the observed and predicted intensity level one or two weeks before.

When the season ended, we evaluated the quality of predictions analysing CISR and ILSR for the full period and for the epidemic period only.

We also calculated MSE and AIC with data from 2015–2016 season for the selected and the four rejected models to evaluate possible differences with respect to the previous season.

**Fig 1:**
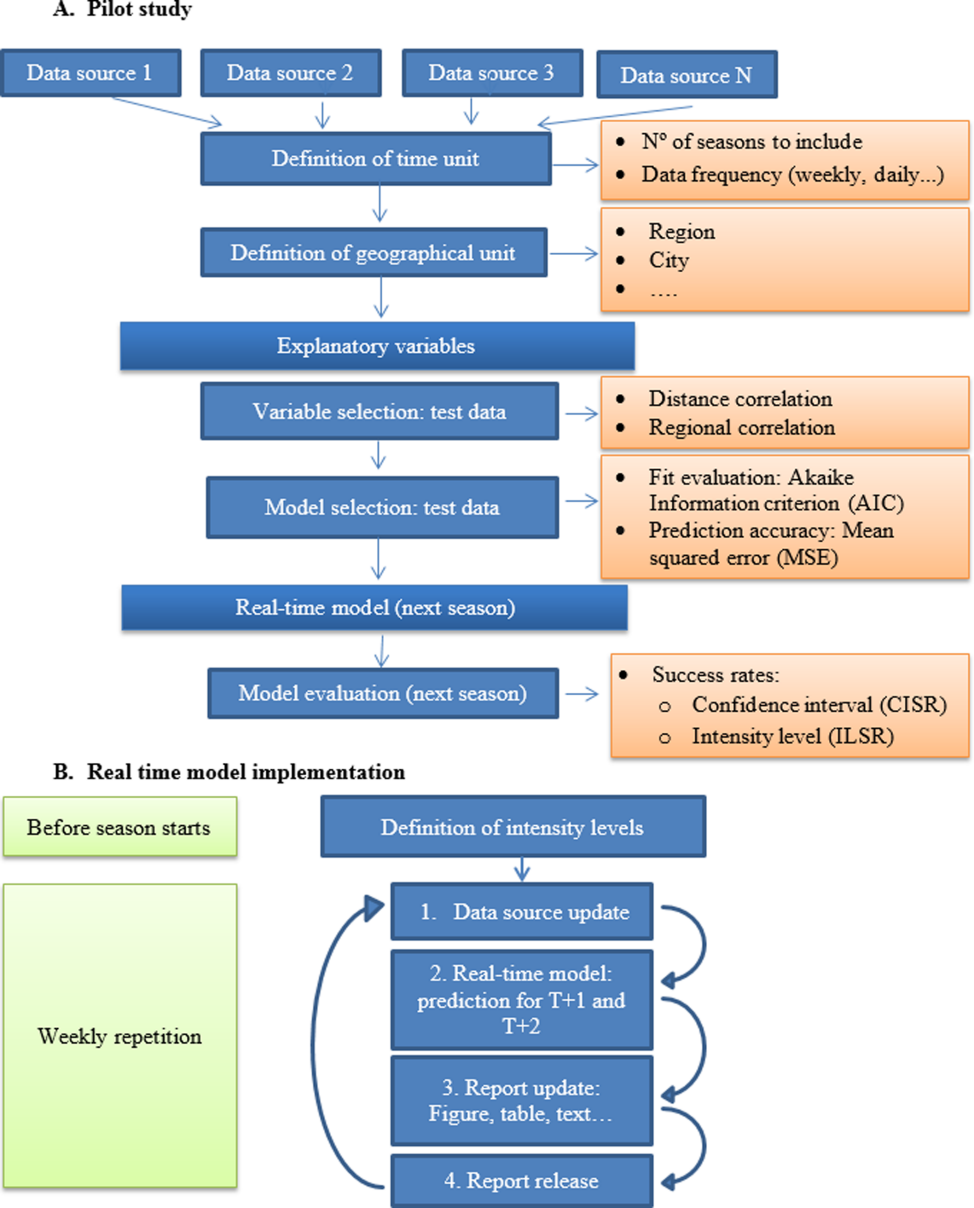
Flow diagram of pilot study (A) and real-time model (B).

The steps of the pilot study and the real-time model implementation are shown in Fig 1.

## Results

### Pilot study

In the pilot study, distance correlation analysis helped to choose, for each data source, the variable that presented the best correlation with the predicted rates at one and two weeks.

These were the simple rate (MDO_ILI) for the MDO source, the global rate (Pidirac) for PIDIRAC source, the isolation percentage (Iso%) for the Laboratory source, the functional temperature (Temp_F) for the Meteocat source and Google Flu Trends for Catalonia (Table 1). Finally, The Google Flu Trends data source was not included in the real-time forecast model due to lack of updates in the 2015–2016 season. For this reason, in the pilot test we repeated the AIC and MSE analysis with and without Google Flu Trend.

**Table 1:** Distance correlations between variables from different data sources in week T and observed MDO rate for week T+1 or T+2. Catalonia 2014–2015.

Data sources (week T)	Distance correlations	
	MDO_ILI (week T+1)	MDO_ILI (week T+2)
**MDO source**		
**1. MDO_ILI**	0.92	0.77
**2. MDO_SIR**	0.90	0.75
**PIDIRAC Source**		
**1. Pidirac**	0.90	0.78
**2. Adult**	0.89	0.77
**3. Child**	0.87	0.77
**4. Baby**	0.87	0.76
**5. Elderly**	0.67	0.57
**Laboatory Source**		
**1. Iso%**	0.78	0.71
**Meteocat source**		
**1. Temp_F**	0.48	0.50
**2. Temp**	0.45	0.45
**3. Irr_F**	0.29	0.37
**4. Irr**	0.29	0.36
**5. Hum_F**	0.19	0.14
**6. Hum**	0.12	0.09
**Google Flu Trends source**		
**1. Catalonia**	0.81	0.85
**2. Valencia**	0.80	0.86
**3. Aragon**	0.77	0.75
**4. Spain**	0.75	0.81

The study of regional distance correlation detected a very strong correlation between regions (> 0.92 for each pair of regions), showing there were not many differences in epidemic trend between regions (Table A in S1 Table). For this reason we include for each region *i* as explanatory variable the MDO_ILI of the region *k* (*k ≠ i*) that present highest correlation with region I (MDO_ILI_NEXT).

So we had X = {MDO_ILI, MDO_ILI_NEXT, Pidirac, Iso%, Temp) as predictor variables for ARIMA, LM and GLS models. For FLM and FGLS models we had X = {MDO_ILI, MDO_ILI_NEXT Pidirac, Iso%) as predictor variables and X(t) = {Temp_F} as functional predictor variable.

The AIC test showed that FGLS is the best fitted model for the entire time series 2010–2015 (146 observations), followed by GLS. The same analysis including the variable Google flu trend shows how improved the model estimation, decreasing AIC (Table B in S1 Table).

Results for MSE analysis are similar to AIC evaluation, confirming that FGLS is the model that best minimizes prediction errors at one and two weeks. (Table 2, S1 Fig).

**Table 2:** Mean squared error (MSE) of prediction rates at one (T+1) and two weeks (T+2) for the five models and the seven regions. Catalonia 2014–2015 and 2015–2016.

	ARIMA				LM				GLS				FLM				FGLS			
	MSE T+1		MSE T+2		MSE T+1		MSE T+2		MSE T+1		MSE T+2		MSE T+1		MSE T+2		MSE T+1		MSE T+2	
	14–15	15–16	14–15	15–16	14–15	15–16	14–15	15–16	14–15	15–16	14–15	15–16	14–15	15–16	14–15	15–16	14–15	15–16	14–15	15–16
**Lleida**	1169	1401	6275	4881	2548	2042	8698	5611	2386	1983	2817	2128	2342	1985	2734	2341	1014	1070	2180	1648
**Tarragona**	487	1032	2079	4103	639	1199	3371	2418	750	1165	918	1246	611	1108	735	1312	236	804	642	1111
**Terres de l'Ebre**	1159	4024	3819	12798	1258	4675	4357	10978	953	5600	1068	6653	1241	4608	1358	5548	420	3640	880	5654
**Girona**	1384	559	5594	2270	1646	695	5548	1226	1268	663	1809	684	1567	583	1978	757	756	490	1852	617
**Catalunya Central**	647	1618	2886	4566	999	1105	3389	1975	820	1511	915	1599	949	1095	1158	1156	300	1116	621	1287
**Alt Pirineu**	1381	1764	2886	4035	1755	1389	3688	1951	2048	2758	2189	3141	1574	1353	1852	1656	1312	1543	1394	1862
**Barcelona**	532	844	2945	2868	1227	519	4358	1116	790	612	991	640	1146	470	1497	585	348	450	875	597
**Total**	**966**	**1606**	**3783**	**5075**	**1439**	**1661**	**4773**	**3611**	**1288**	**2042**	**1530**	**2299**	**1347**	**1600**	**1616**	**1908**	**627**	**1302**	**1206**	**1825**
**Total with GFT**	**742**	**-**	**2628**	**-**	**1008**	**-**	**3031**	**-**	**991**	**-**	**1133**	**-**	**1002**	**-**	**1121**	**-**	**532**	**-**	**786**	**-**

### Real-time model

During the 2015–2016 season, the FGLS real-time model was applied weekly from week 40 of 2015 to week 20 of 2016 (34 weeks).

**Fig 2:**
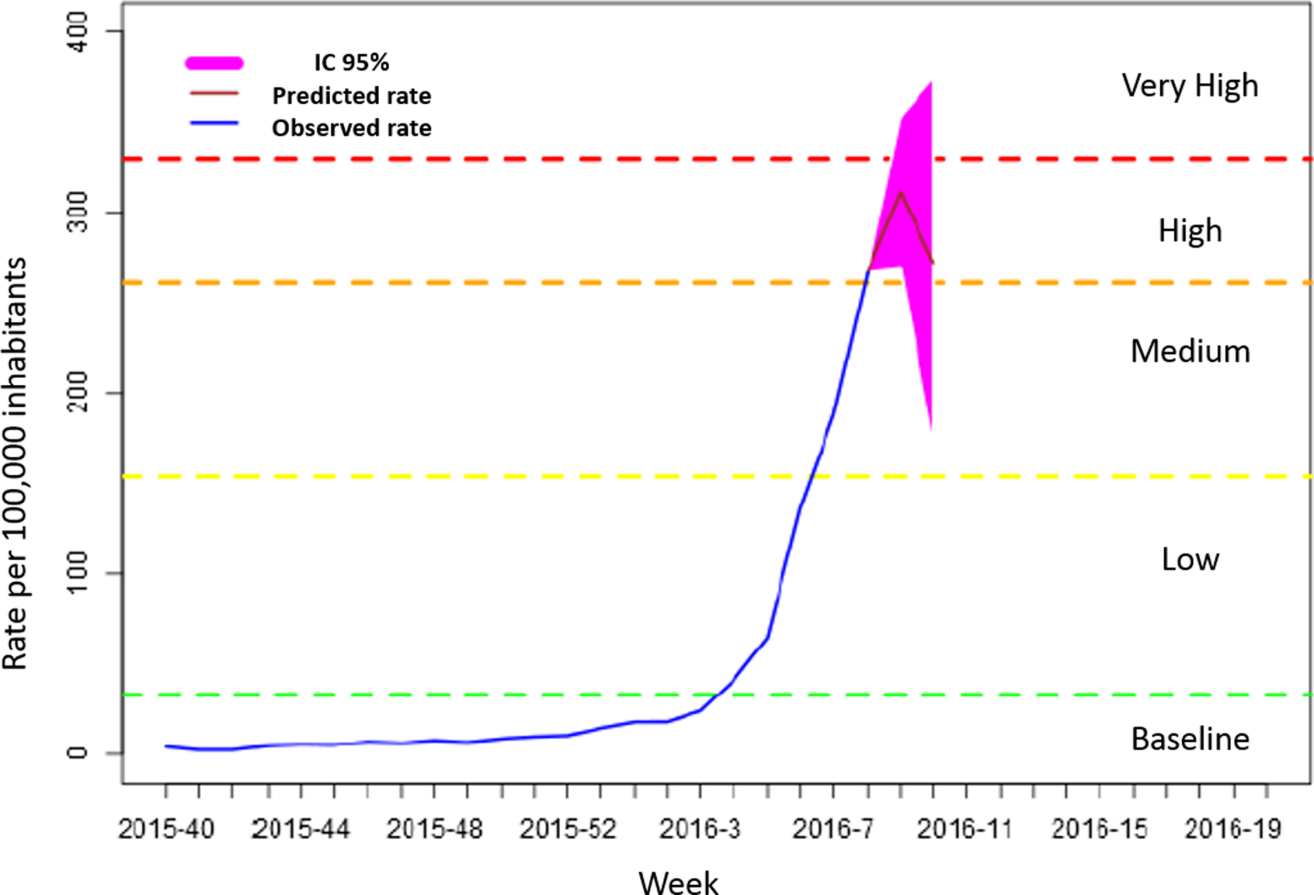
Global observed epidemic curve for week 8 (T) and predicted rates with 95% CI for week 9 (T+1) and 10 (T+2). The blue curve represent the observed incidence rates from week 40 of 2015 to week 8 of 2016.The red curve represent the predicted incidence rates for week 9 and 10. The pink area represent the 95%CI for predictions. Horizontal lines represent the epidemic intensity levels of influenza for 2015–2016 calculated with MEM.

**Fig 3:**
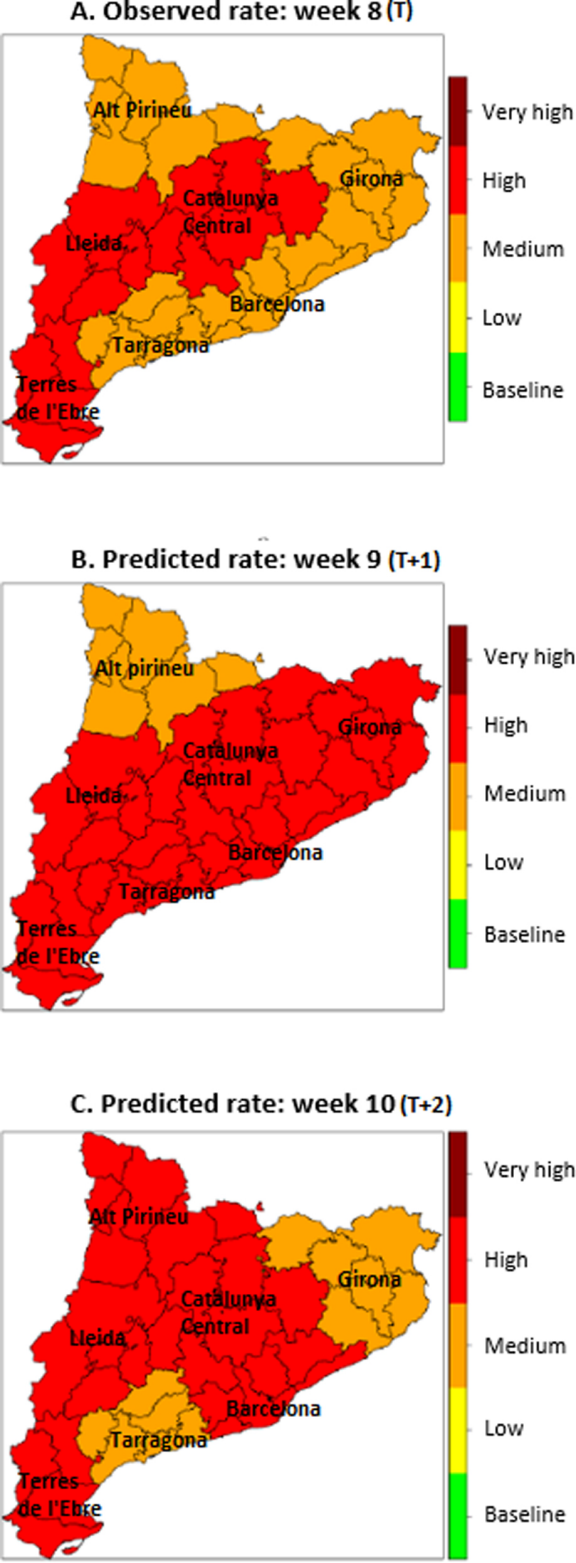
Observed epidemic intensity levels for week 8 (A) for each region and predicted intensity for week 9 (B) and 10 (C). (A) Represent Catalonia map in which the observed intensity level during week 8 of 2016 (T) for each region are illustrated: for example, Alt Pirineu presented in week 8 a medium epidemic intensity level of influenza. (B) Show in the same map the predicted intensity level for each region for week 9 (T+1): for example, a high intensity level of influenza was predicted for Barcelona for week 9. (C) Show in the same map the predicted intensity level for each region for week 10 (T+2).

A report was published weekly at the official web page representing observed and predicted rates in different formats. Figs 2 and 3 represent examples of the graphics created for the report of week 8 of 2016 showing the global observed epidemic curve and intensity levels for each region for that particular week and the predictions for the next two weeks.

The one week prediction incidence curve (T+1) included 33 observations (from week 41 of 2015 to week 20 of 2016) and the two week prediction curve (T+2) included 32 observations (from week 42 of 2015 to week 20 of 2016).

The epidemic intensity levels calculated using the MEM were: baseline (no activity) between 0 and 33.01 cases per 100,000 inhabitants, low epidemic level between 33.01 and 153.84, medium epidemic level between 153.84 and 261.18, high epidemic level between 261.18 and 330.02, very high epidemic level above 330.02. The epidemic period was defined as any rate > 33.01 cases per 100,000 inhabitants.

The epidemic curve of the 2015–2016 season presented a regular pattern, with a baseline level from week 40 of 2015 to week 3 of 2016 and from week 15 to week 20 of 2016. There was an epidemic situation between weeks 4 and 14, with a peak of 323 cases per 100,000 inhabitants at week 10, representing a high epidemic level according to the MEM levels calculated, but without reaching the highest epidemic level of 330.02.

**Fig 4:**
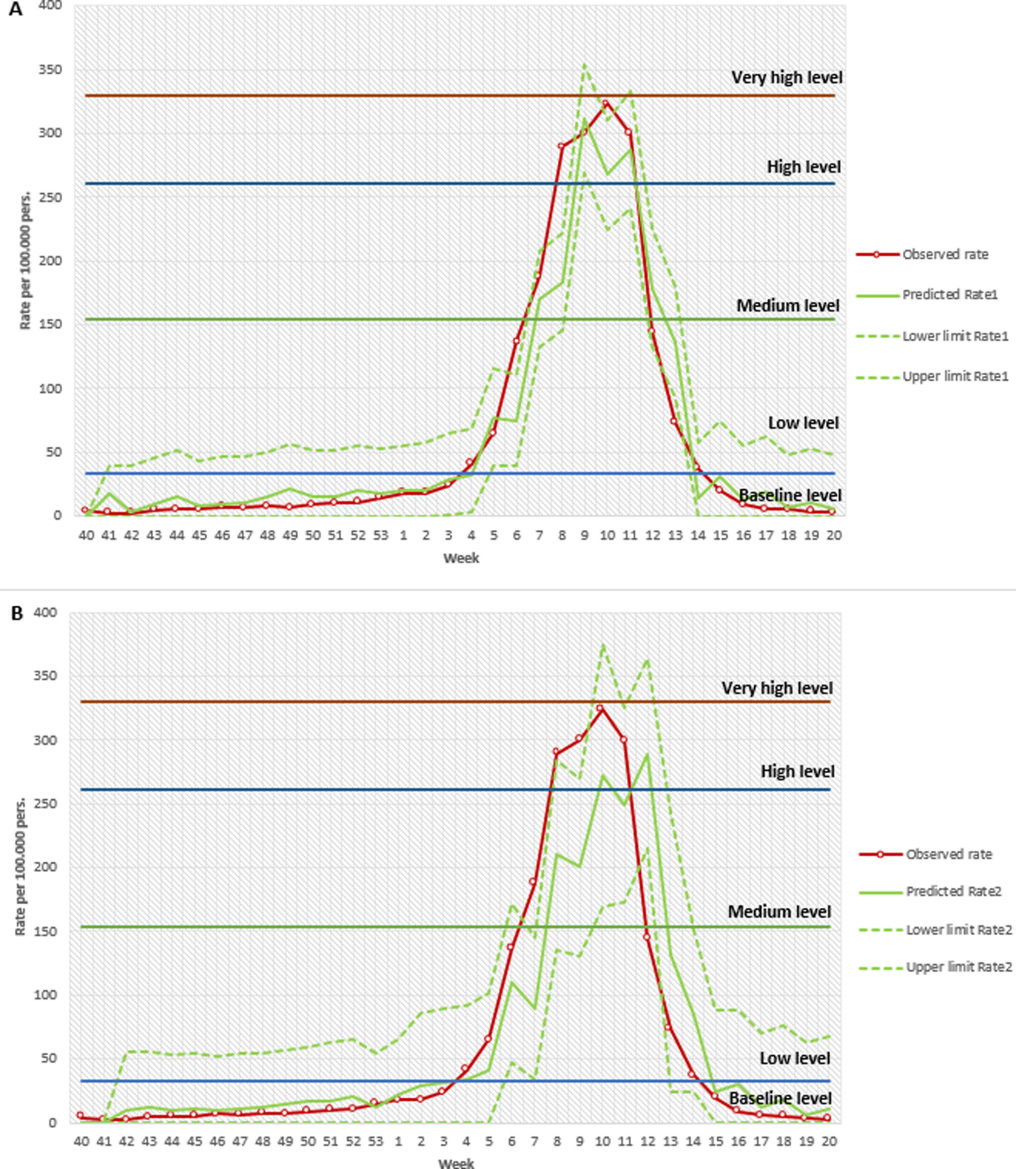
Observed curve vs. Predicted curve one and two week previously (T+1 and T+2, respectively), with 95% CI. Catalonia 2015–2016. (A) Observed curve is compared to predicted curve and corresponding 95% CI one week before. Horizontal lines represents the epidemic intensity levels for 2015–2016 season. (B) Observed curve is compared to predicted curve and corresponding 95% CI two weeks before. Horizontal lines represents the epidemic intensity levels of influenza for 2015–2016 calculated with MEM.

The T+1 prediction curve estimated the epidemic period between weeks 5 and 13 of 2016 and the T+2 prediction curve estimated the epidemic period between weeks 4 and 14, as the observed rate (Fig 4).

The MSE of the FGLS model in this season were worse than for the pilot study season (1302 for T+1 and 1825 for T+2) but remained better than the four rejected models (Table 2).

Globally, 85.3% of all season observations fell within the CI predicted a week previously. The same percentage for T+2 was higher (87.1%) both globally and by region (Table 3). This is because the T+2 CI were wider than for T+1 (Fig 4). For the epidemic period, the success rate decreased to 58.4% for T+1 and 63.6% for T+2 (Table 3).

**Table 3:** Confidence interval success rate (CISR) and intensity level success rate (ILSR) for prediction rates one (T+1) and two (T+2) weeks previously for the seven regions. Season 2015–2016.

	Confidence Interval success rate				Intensity Level success rate			
	Total season		Epidemic period		Total season		Epidemic period	
	CISR T+1 (%)	CISR T+2 (%)	CISR T+1 (%)	CISR T+2 (%)	ILSR T+1 (%)	ILSR T+2 (%)	ILSR T+1 (%)	ILSR T+2 (%)
**Lleida**	87.1	90.3	63.6	72.7	74.2	83.9	54.5	63.6
**Tarragona**	87.1	87.1	63.6	63.6	83.9	83.9	63.6	63.6
**Terres de l'Ebre**	77.4	83.9	36.4	54.5	80.6	74.2	54.5	54.5
**Girona**	83.9	90.3	54.5	72.7	87.1	87.1	72.7	72.7
**Catalunya Central**	90.3	87.1	72.7	63.6	80.6	80.6	54.5	54.5
**Alt Pirineu**	83.9	83.9	54.5	54.5	80.6	77.4	54.5	45.5
**Barcelona**	87.1	87.1	63.6	63.6	93.5	87.1	81.8	72.7
**Total**	**85.3**	**87.1**	**58.4**	**63.6**	**82.9**	**82.0**	**62.3**	**61.0**

Analysis of the intensity level success rate showed that predicted intensity level matched the observed level in 82.9% of cases for T+1 and in 82% of cases for T+2. Focusing only on the epidemic period, the success rates were 62.3% and 61%, respectively (Table 3). Highest matching rate (92.4%) was observed at the baseline level of intensity and as intensity increased matching rate decreased. At the "very high" level the matching rate was of 71.4% for T+2 (Table 4).

**Table 4:** Intensity level success rate (percentage) for prediction rates one (T+1) and two (T+2) weeks previously for each intensity level.

	Intensity Level Success Rate		
Intensity levels	N	ILSR T+1 (%)	ILSR T+2 (%)
**Baseline**	145	92.4	91.7
**Low**	32	71.9	78.1
**Medium**	23	60.9	43.5
**High**	10	50.0	50.0
**Very High**	7	57.1	71.4
**Total**	**217**	**82.9**	**82.0**

## Discussion

The model described is an attempt to provide a forecast of the influenza incidence rate, taking into account the epidemic curve of the previous five years and its relationship with the explanatory variables. Therefore, the prediction offers a predicted value and an interval (with 95% confidence) within future observation should be if influenza behaved normally, given the background. When the observed value was outside the predicted intervals one or two weeks earlier, there are "deviations", indicating that there are other factors, not described by the known data sources, which affect the prediction. Uncertainty is impossible to quantify and is always be present and limits the effectiveness of predictions.

The data sources used are those routinely used by any influenza surveillance system in any country or region, making this type of methodology easily exportable to other regions and even other diseases with a similar pattern.

The decision to make a pilot test to define the variables and the model to be used in the following season was taken to avoid repeating each week the model and variables selection process, as a matter of practicality loading the model and uniformity of the results.

The AIC evaluation and predictive curve in pilot test (S1 Table, S1 Fig) demonstrates that GLS models adjust the linear trend equally as well as the LM, but provided the possibility of overcoming the problem of correlated errors by applying a time sequence AR(p = 1). ARMA (1,1) model also shows good AIC results thanks to a constant seasonal variability in the time series. Instead the rigid assumptions of a regular trend in epidemic curve every season and a constant mean and variance limit the quality of estimations compared to GLS, especially for T+2.

Moreover, models that include the functional component (FLM, FGLS) estimate better than their corresponding models without functional component (LM, GLS), as expected seeing the correlation results from meteorological source. [25]

We can observe a solid difference between AIC and MSE results for GLS model. AIC show that GLS is similar to FGLS and better than the others 3 models, but in MSE analysis predictions errors seems to be almost double than FGLS and higher than ARIMA for T+1. It could be explicated because the gls() function in nlme R-package only take into account the correlation structure of estimation but don’t include the estimated dependence of errors. This problem is solved for FGLS in the fregre.gls () from fda.usc R-package.

The results for the 2015–2016 season showed that the MSEs for week T+1 increased almost two-fold during this period compared with pilot test (Table 3). This is mainly due to a different pattern of the epidemic curve compared with the past. In 2013–2014 and 2014–2015 seasons, epidemic activity began 2–3 weeks earlier, with a shorter duration, and peaking higher than the 2015–2016 season. These three factors, start, duration and peaks, are key to making predictions work and when any factor varies widely with respect to the past, the prediction is worse.

Analysis of the differences between the success rates of T+1 and T+2 predictions shows there was not much difference as in the MSE scores: this is mainly due to the longer time horizon, which implies a wider confidence interval for T+2 predictions.

Another important aspect is the difference between epidemic and non-epidemic periods and between lower and higher intensity levels. Lower success rates were associated with steep increases in the seasonal epidemic activity curve and continues to decrease during the epidemic period, when the prediction is harder to make. The results for T+2 are similar to T+1, excluding the "very high" level where the percentage of success was 71.4%, but this could be because of a small number of observations at this level of activity (Table 4).

Given the results, it does not seem wise to extend the prediction to three weeks or one month with these explanatory variables, taking into account that for T+2 MSE is very high and CI very wide.

Future improvements in the model will include the identification, within the available data sources, of other variables that would improve the prediction.

For example, Google flu trend presented a very good correlation in the pilot test and predictions got better including this factor in the model, with a sensible decrease of the prediction errors, and it would be important to find a variable that provides similar information about population consultations on influenza. In this sense, there is a free telephone service for the citizens of Catalonia, from which the weekly volume of public consultations on influenza could probably be extracted, adding useful information.

Thus, it would be interesting to replace meteorological observed data with forecast data in order to predict in advance abrupt changes in temperatures which might influence the influenza rate. The circulating seasonal influenza strain is another factor that could affect the duration and peaks of the seasonal epidemic, and its inclusion might also improve the prediction.

In conclusion, the model was shown to be useful but could be improved by further use.

## Supporting information

S1 Appendix Mathematical equations for the 5 selected models. (DOCX)

S1 Table Distance correlation between sanitary regions in 2014–2015 season (A) and AIC for the 5 fitted models for 2010–2015 and 2010–2016 (B). (DOCX)

S1 Fig Estimation of the prediction one week previously (T+1) for the 5 different models, season 2014–2015 and 2015–2016. (PDF)
