# Supplementary material for: Real-time predictive seasonal influenza model in Catalonia, Spain
Source: PLoS One. 2018 Mar 7;13(3):e0193651. doi: 10.1371/journal.pone.0193651 (PMC5841785; doi:10.1371/journal.pone.0193651)
Supplement: S1 Appendix — (DOCX) [file pone.0193651.s001.docx]

**S1 Appendix. Mathematical equations for the 5 selected models.**

**Autoregressive Moving Average model (ARMA)**

ARMA (1,1): $Y= \alpha+ <X,\beta>+ \varepsilon$

$Y= \alpha+ \sum_{i=1}^{p} \Phi_{i}X_{t-i}+\sum_{i=1}^{q} \theta_{i}\varepsilon_{t-i}$ and $\Phi X_{t}= \theta\varepsilon_{t}$

Where $\alpha$ is the intercept,

$\Phi{=\left\{ \Phi_{i} \right\}}_{i=1}^{p}$ is the vector of p parameters of the autoregressive part (AR),

${\theta=\left\{ \theta_{i} \right\}}_{i=1}^{q}$is the vector of q parameters of the moving average part (MA).

**Linear Model (LM)**

LM: $Y= <X,\beta>+ \varepsilon$

$Y= X^{'}\beta+ \varepsilon, \varepsilon\sim N\left( 0, \sigma^{2} \Sigma\right)$,

Where $\Sigma=I=$ $\left( \begin{matrix} 1 & 0 & 0 \\ 0 & 1 & 0 \\ 0 & 0 & 1 \end{matrix} \right)$

$\hat{\beta}=\left( X^{'}X \right)^{-1}X^{'}y$

$$Var(\hat{\beta})={\sigma^{2}\left( X^{'}X \right)}^{-1}$$

**Generalized leas Squared model (GLS)**

GLS: $Y=<X,\beta>+ \varepsilon$

$$Y= X^{'}\beta+ \varepsilon, \varepsilon\sim N\left( 0, \sigma^{2}\Sigma\right)$$

Where $\Sigma=W^{-1}=$ $\left( \begin{matrix} 1 & \Phi& \Phi^{2} \\ \Phi& 1 & \Phi\\ \Phi^{2} & \Phi& 1 \end{matrix} \right)$

$\hat{\beta}=\left( X^{'}WX \right)^{-1}X^{'}Wy$

$$Var(\hat{\beta})={\sigma^{2}\left( X^{'}WX \right)}^{-1}$$

**Functional** **Linear Model (FLM)**

FLM: $Y=<X\left( t \right),\beta\left( t \right)>+ \varepsilon$

$$Y= X^{'}\beta+\int X\left( t \right)\beta\left( t \right)dt+ \varepsilon, \varepsilon\sim N\left( 0, \sigma^{2}\Sigma\right)$$

Where $\Sigma=I=$ $\left( \begin{matrix} 1 & 0 & 0 \\ 0 & 1 & 0 \\ 0 & 0 & 1 \end{matrix} \right)$

$\hat{\beta}=\left( X^{'}X \right)^{-1}X^{'}y$

$$Var(\hat{\beta})={\sigma^{2}\left( X^{'}X \right)}^{-1}$$

**Functional Generalized leas Squared model (FGLS)**

FGLS: $Y=<X(t),\beta(t)>+ \varepsilon$

$Y= \int X\left( t \right)\beta\left( t \right)dt+ \varepsilon, \varepsilon\sim N\left( 0, \sigma^{2}\Sigma\right)$

Where $\Sigma=W^{-1}=$ $\left( \begin{matrix} 1 & \Phi& \Phi^{2} \\ \Phi& 1 & \Phi\\ \Phi^{2} & \Phi& 1 \end{matrix} \right)$

$\hat{\beta}=\left( X^{'}WX \right)^{-1}X^{'}Wy$

$$Var(\hat{\beta})={\sigma^{2}\left( X^{'}WX \right)}^{-1}$$
