# Supplementary material for: Real-time predictive seasonal influenza model in Catalonia, Spain
Source: PLoS One. 2018 Mar 7;13(3):e0193651. doi: 10.1371/journal.pone.0193651 (PMC5841785; doi:10.1371/journal.pone.0193651)
Supplement: S1 Table — (DOCX) [file pone.0193651.s002.docx]

**S1 Table. Distance correlation between sanitary regions in 2014-2015 season (A) and AIC for the 5 fitted models for 2010-2015 and 2010-2016 (B).**

**A**

|  | Lleida | Tarragona | Terres de l'Ebre | Girona | Catalunya Central | Alt Pirineu | Barcelona |
| --- | --- | --- | --- | --- | --- | --- | --- |
| Lleida | 1 | 0,96 | 0,95 | 0,96 | 0,97 | 0,93 | 0,98 |
| Tarragona | 0,96 | 1 | 0,96 | 0,98 | 0,98 | 0,94 | 0,97 |
| Terres de l'Ebre | 0,95 | 0,96 | 1 | 0,96 | 0,95 | 0,93 | 0,94 |
| Girona | 0,96 | 0,98 | 0,96 | 1 | 0,98 | 0,94 | 0,97 |
| Catalunya Central | 0,97 | 0,98 | 0,95 | 98 | 1 | 0,94 | 0,98 |
| Alt Pirineu | 0,93 | 0,94 | 0,93 | 0,94 | 0,94 | 1 | 0,93 |
| Barcelona | 0,98 | 0,97 | 0,94 | 0,97 | 0,98 | 0,93 | 1 |

**B**

|  | **ARIMA** | | **LM** | | **GLS** | | **FLM** | | **FGLS** | |
| --- | --- | --- | --- | --- | --- | --- | --- | --- | --- | --- |
|  | **AIC** | | **AIC** | | **AIC** | | **AIC** | | **AIC** | |
|  | **2010-2015** | **2010-2016** | **2010-2015** | **2010-2016** | **2010-2015** | **2010-2016** | **2010-2015** | **2010-2016** | **2010-2015** | **2010-2016** |
| **Lleida** | 1354 | 1406 | 1480 | 1515 | 1358 | 1412 | 1479 | 1514 | 1360 | 1408 |
| **Tarragona** | 1364 | 1396 | 1417 | 1424 | 1349 | 1387 | 1420 | 1424 | 1347 | 1377 |
| **Terres de l'Ebre** | 1356 | 1465 | 1409 | 1509 | 1305 | 1452 | 1410 | 1510 | 1306 | 1448 |
| **Girona** | 1316 | 1338 | 1350 | 1350 | 1260 | 1299 | 1352 | 1345 | 1258 | 1286 |
| **Catalunya Central** | 1297 | 1364 | 1367 | 1393 | 1289 | 1357 | 1368 | 1396 | 1289 | 1355 |
| **Alt Pirineu** | 1398 | 1438 | 1401 | 1430 | 1383 | 1428 | 1396 | 1424 | 1378 | 1418 |
| **Barcelona** | 1186 | 1269 | 1317 | 1334 | 1195 | 1268 | 1315 | 1329 | 1191 | 1261 |
| **Total** | **1324** | **1382** | **1391** | **1422** | **1306** | **1372** | **1391** | **1420** | **1304** | **1365** |
| **Total with GFT** | **1324** | **-** | **1351** | **-** | **1281** | **-** | **1355** | **-** | **1284** | **-** |
