# Supplementary figures and images for: Real-time predictive seasonal influenza model in Catalonia, Spain
Source: PLoS One. 2018 Mar 7;13(3):e0193651. doi: 10.1371/journal.pone.0193651 (PMC5841785; doi:10.1371/journal.pone.0193651)

**S1 Fig. Estimation of the prediction one week previously (T+1) for the 5 different models, season 2014-2015 and 2015-2016.**

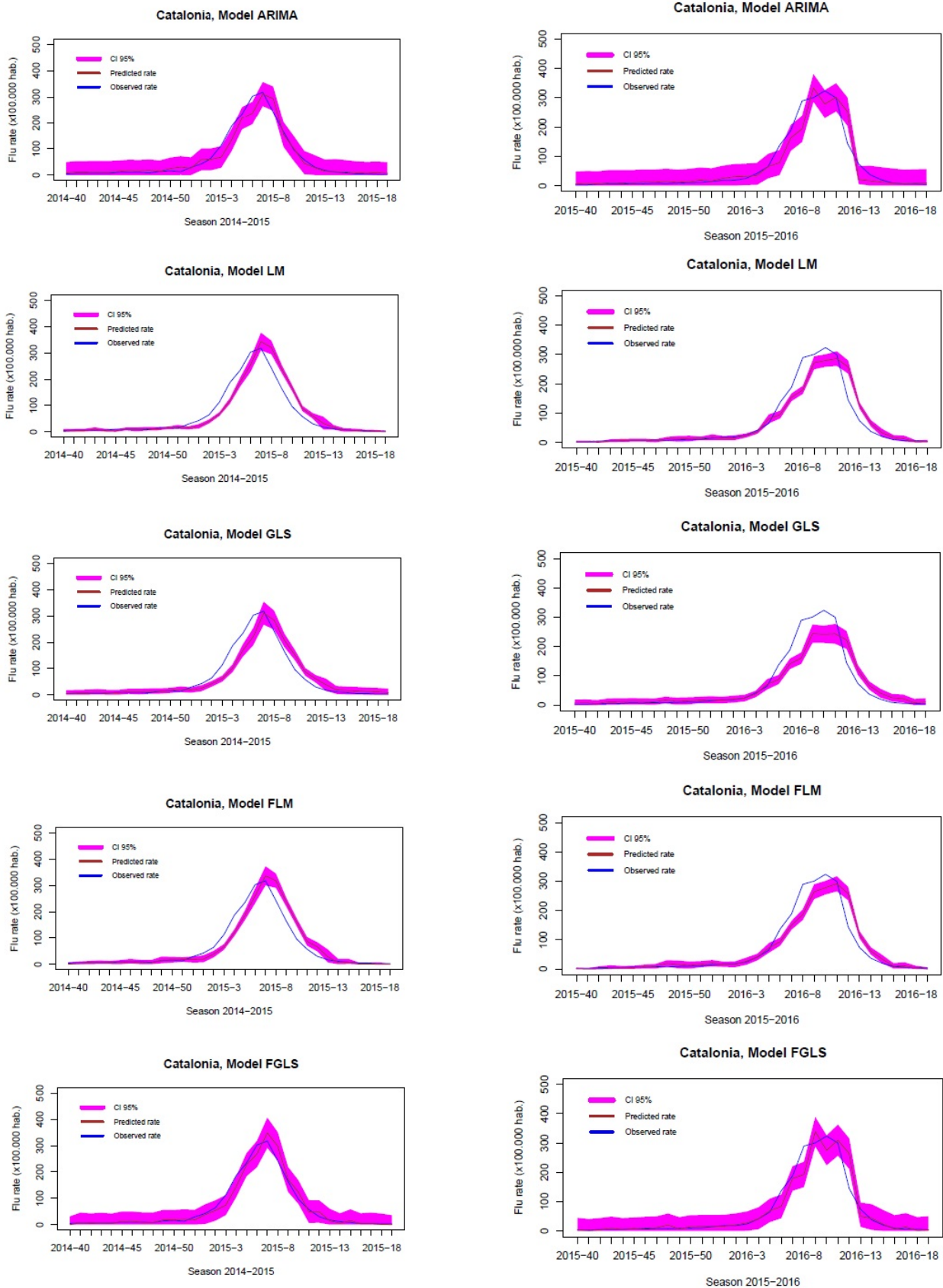

Supplement: S1 Fig — (PDF) [file pone.0193651.s003.pdf]
